# Oseltamivir-Resistant Pandemic (H1N1) 2009 in Patient with Impaired Immune System

**DOI:** 10.3201/eid1607.091579

**Published:** 2010-07

**Authors:** Grant A. Hill-Cawthorne, Silke Schelenz, Matthew Lawes, Samir Dervisevic

**Affiliations:** Author affiliations: University of East Anglia, Norwich, UK; and; Norfolk and Norwich University Hospital, Norwich

**Keywords:** viruses, influenza A, H1N1, pandemic, oseltamivir, immunocompromised, zanamivir, respiratory infections, Hodgkin disease, letter

**To the Editor:** We detail the development of oseltamivir-resistant pandemic (H1N1) 2009 in a chronically immunocompromised patient and the pitfalls encountered when treating such patients with neuraminidase inhibitors. On August 6, 2009, a 56-year-old man was seen in the emergency room of a local hospital with a 24-hour history of fever, myalgia, coryzal symptoms, and cough. He was on day 3 of a postexposure course of oseltamivir (75 mg 1×/d); influenza A had been presumptively diagnosed for his wife after she had similar symptoms.

The patient’s medical history showed grade IVB nodular sclerosing Hodgkin lymphoma, which had been diagnosed in 2001. Lymphoma was initially treated with chemotherapy, but relapse required autologous peripheral stem cell transplantation in July 2005. Further relapses in 2006 and 2007 were treated with radiotherapy and chemotherapy, respectively, before the patient underwent an allogeneic peripheral stem cell transplantation in July 2008. This treatment was complicated by graft-versus-host disease, and the patient required ongoing immunosuppression.

When hospitalized, the patient was being treated with cyclosporine A (50 mg/d) and prednisolone (20 mg/d). Physical examination showed a temperature of 39°C and wheezing from the left lung. Initial tests showed a neutrophil count of 2.02 × 10^9^/L, a lymphocyte count of 0.87 × 10^9^/L, and a C-reactive protein level of 33 mg/L. He was started on piperacillin–tazobactam and gentamicin, and oseltamivir was increased to the treatment dose of 75 mg 2×/d. A nasopharyngeal aspirate collected on August 7 contained pandemic (H1N1) 2009 viral RNA by real-time PCR for generic influenza A (*1*) and capillary sequencing for subtype H1N1 (testing by Micropathology Ltd, Coventry, UK). By August 9, the patient was still febrile, and zanamivir (10 mg 2×/d) was started. Oseltamivir was given for a total of 7 d and zanamivir for 3 d.

Nose and throat swabs taken on August 21 still contained pandemic (H1N1) 2009 viral RNA. Real-time PCR and pyrosequencing demonstrated a histidine-to-tyrosine substitution (H275Y) in the neuraminidase gene associated with oseltamivir resistance (Respiratory Virus Unit, Centre for Infections, Health Protection Agency; methods not in public domain). A mixture of wild-type and resistant virus was present (A. Lackenby, pers. comm.). The sample from August 7 did not contain this mutation, suggesting a de novo H275Y substitution secondary to oseltamivir use.

The patient improved and was discharged on August 23 but returned for treatment on September 7 with worsening fever and cough. Nose and throat swabs obtained on September 11 were PCR negative, but follow-up samples on September 25 and October 1 contained detectable pandemic (H1N1) 2009 viral RNA. Because virus isolation was not performed, true infectivity remains unresolved, but intermittent detection suggests ongoing replication, such as that seen in other immunocompromised patients (*2**,**3*).

By February 3, 2010, a total of 225 cases of oseltamivir-resistant pandemic (H1N1) 2009 had been identified worldwide; a high proportion of cases were in immunocompromised persons (*4*). A minority of these mutations were detected in treatment-naive patients. Immunocompromised, particularly lymphopenic, patients shed virus for prolonged periods leading to longer treatment courses and viral shedding reviving on termination of treatment. Viral shedding for up to 18 months has been reported for seasonal influenza, which has important implications for infection control (*5*). Our patient demonstrated that a single PCR-negative test does not reliably determine the end of viral shedding, which continued despite co-treatment with 2 neuraminidase inhibitors. Neuraminidase inhibitors interfere with the release of progeny influenza virus from their infected host cells. Effective treatment depends partially on immune system destruction of the foci of infection (*6*), or potential persistent viral particles can be released as soon as oseltamivir therapy is stopped. The low genetic barrier to oseltamivir means that resistance is a likely consequence of monotherapy in immunocompromised patients.

Concern about oseltamivir resistance has led to issuance of additional guidelines, especially in light of the transmission of resistant virus between immunocompromised patients on hospital wards in the United States and Wales (*7**,**8*). This finding suggests that immunocompromised patients should be treated with oseltamivir and zanamivir, or with zanamivir alone, for a minimum of 10 d. Patients should be retested for ongoing viral secretion every 5 d and negative results confirmed with a follow-up sample after 48 h. Classic virus isolation in addition to molecular methods may also identify potentially infectious patients.

Prophylactic neuraminidase inhibitor use in such patients also needs to be addressed. Resistance is more likely with the reduced prophylactic dose of oseltamivir and is more likely to be a problem in immunocompromised patients. Zanamivir is now the drug of choice for prophylaxis for such patients, although some experts propose no prophylaxis and instead early treatment after symptom onset (*9*).

Immunocompromised patients are more likely to shed virus for prolonged periods and are more likely to develop oseltamivir-resistance, especially when this drug is used as monotherapy. Further clinical experience and trials will support or refute newer guidelines on the management of pandemic (H1N1) 2009 in such patients.
